# Supplementary material for: The Peanut Skin Procyanidins Attenuate DSS-Induced Ulcerative Colitis in C57BL/6 Mice
Source: Antioxidants (Basel). 2022 Oct 25;11(11):2098. doi: 10.3390/antiox11112098 (PMC9686776; doi:10.3390/antiox11112098)
Supplement: Supplementary file 1 [file antioxidants-11-02098-s001.zip › antioxidants-1905679-supplementary.pdf]

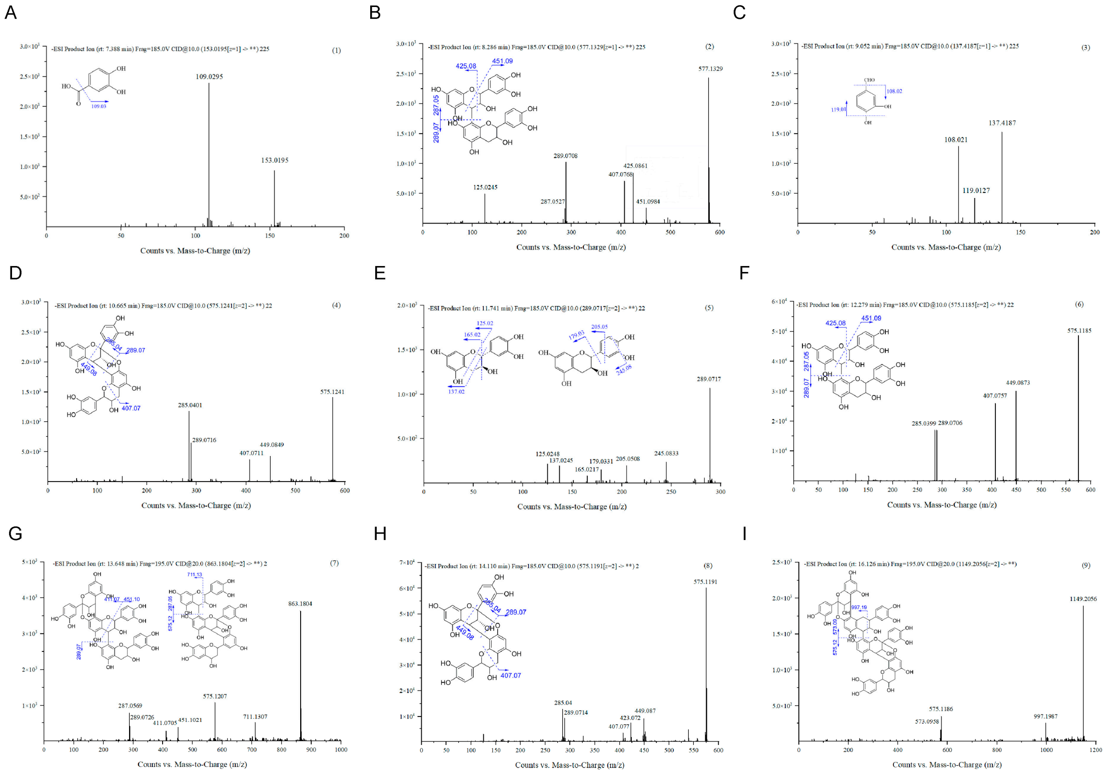

Figure S1. Composition of PSPc. (**A**)MS/MS spectra of compounds 1 in negative ion mode; (**B**)MS/MS spectra of compounds 2 in negative ion mode; (**C**)MS/MS spectra of compounds 3 in negative ion mode; (**D**)MS/MS spectra of compounds 4 in negative ion mode; (**E**)MS/MS spectra of compounds 5 in negative ion mode; (**F**)MS/MS spectra of compounds 6 in negative ion mode; (**G**)MS/MS spectra of compounds 7 in negative ion mode; (**H**)MS/MS spectra of compounds 8 in negative ion mode; (**I**)MS/MS spectra of compounds 9 in negative ion mode.
